# Scaling PatientsLikeMe via a “Generalized Platform” for Members with Chronic Illness: Web-Based Survey Study of Benefits Arising

**DOI:** 10.2196/jmir.9909

**Published:** 2018-05-07

**Authors:** Paul Wicks, Eileen Mack Thorley, Kristina Simacek, Christopher Curran, Cathy Emmas

**Affiliations:** ^1^ PatientsLikeMe Cambridge, MA United States; ^2^ AstraZeneca UK Ltd Luton United Kingdom

**Keywords:** personal health records, personal monitoring, technology, health care, self-help devices, personal tracking, social support, online support group, online health community

## Abstract

**Background:**

Launched in 2006 for patients with amyotrophic lateral sclerosis, PatientsLikeMe is an online community offering patient-reported outcomes, symptom tracking, and social features. Every member of the site can see all the data reported by every other member, view aggregated reports, identify “patients like them,” and learn about treatment options in order to live better with their condition. In previous studies, members reported benefits such as improved condition knowledge, increased medication adherence, and better management of side effects. However, the site evolved in 2011 from condition-specific “vertical” communities consisting only of people with the same disease to a “generalized platform,” in which every patient could connect with every other patient regardless of condition and with generic, rather than condition-specific, data tools. Some, but not all, communities received further custom tracking tools.

**Objective:**

We aimed to understand (1) whether members of PatientsLikeMe using the generalized platform still reported similar benefits and (2) assess factors associated with benefits, such as community customization, site use, and patient activation.

**Methods:**

A cross-sectional retrospective custom survey was fielded to 377,625 members between 2016 and 2017 including the Patient Activation Measure (PAM). A benefit index was developed for comparability across conditions.

**Results:**

The invitation was viewed by 26,048 members of whom 11,915 did not respond, 5091 opted out, 1591 provided partial data, and 17 were screened out. Complete responses were received from 7434 participants. Users perceived greatest benefit in understanding how their condition may affect them (4530/6770, 66.91% participants, excluding “does not apply” answers), understanding what might help them live better with their condition (4247/6750, 62.92%), which treatments were available (4143/6898, 60.06%), understanding treatment side effects (4182/6902, 60.59%), and important factors in making treatment decisions (3919/6813, 57.52%). The benefit index was 29% higher for the “most activated” patients (PAM level 4 vs PAM level 1; relative risk [RR]=1.29, *P*<.001), 21% higher for conditions with some community customization versus none (RR=1.21, *P*<.001), and 11% higher in those using the site most often versus least (RR=1.11, *P*<.001).

**Conclusions:**

Members of the generalized platform reported a range of benefits related to improved knowledge and understanding of their condition and treatment management. Condition-specific customization may improve their experience still further. Future studies will explore longitudinal changes to patient activation.

## Introduction

### History of Online Communities for Chronic Illness

Online communities for chronic illnesses have existed since the early 1980s through email list-servs, USENET, and online bulletin boards such as “The WELL” [1], Association of Online Cancer Resources [2], Yahoo! Groups [3], and Braintalk [4]. These emphasized lengthy and anonymous text discussions, making it hard to extract useful information as the communities grew. Later “Web 2.0” technologies and mainstream social networking sites such as Facebook and Twitter grew in popularity [5], meeting patients and caregivers where they were already engaging. However, the public nature of these newer sites may have made users more cautious about sharing health information [6,7].

PatientsLikeMe is an online community that allows members to find other patients like them, share and track their health data over time, and contribute to scientific research [8]. As a for-profit company, PatientsLikeMe works with pharmaceutical and biotechnology companies to share aggregated outcomes data, patient treatment evaluations, and to field surveys to help improve partner services and support patient centricity efforts [9]. Some question whether there is equipoise in the benefits accrued to participant members and the revenue collected by the company [10-12].

Many studies have documented the effect of online health community participation on feelings of empowerment and perceived social support [3,13]. However, systematically studying the effects of such communities on their members has been challenging [14]. The member populations are highly self-selecting, research funding is minimal [3], many users are “lurkers” [15], the most salient experiences are often qualitative, defining scope can be challenging [16], and sample sizes are small and time-limited [17]. By the time a peer-reviewed scientific study has been published, the community itself may have evolved, emptied, or shut down [18].

### Early History of PatientsLikeMe

PatientsLikeMe launched in 2006 for patients with amyotrophic lateral sclerosis (ALS). Over the next 5 years, the site created distinct “vertical” communities. Members could only belong to one at a time, they could not see data from other communities, and there was no ability to add comorbid conditions. Between 2006 and 2011, nine vertical communities were developed for people with multiple sclerosis (MS), Parkinson's disease, HIV, a range of mood disorders, progressive supranuclear palsy, multiple system atrophy, fibromyalgia, myalgic encephalomyelitis / chronic fatigue syndrome, and organ transplants.

A retrospective survey among members of the first six vertical communities reported a number of benefits including: learning about symptoms they had experienced (952/1323, 71.96% rated “very helpful” or “moderately helpful”), understanding side effects of treatment (757/1323, 57.21%), and finding another patient like them on the same treatment (559/1323, 42.25%) [19]. Members reported other changes, such as deciding to start a new medication (496/1323, 37.49%), change their medication dosage (336/1323, 25.39%), or stopping a medication (290/1323, 21.92%). A subset (151/1320, 11.44%) reported changing their physician as a result of using the site. A subsequent survey in epilepsy had similar results, with the additional finding of a dose-effect curve between the number of benefits experienced and the number of social ties made on the site [20].

However, these studies shared limitations of self-selecting populations, being conducted internally, a lack of validated instruments, and a lack of preplanned analyses. A subsequent academic collaboration with the University of California, San Francisco, and the US Department of Veterans Affairs found significant benefits for veterans with epilepsy after 6 weeks of site use. This latter study used validated measures of self-management and self-efficacy in a pre-post design with prespecified analyses [21].

### The Generalized Platform

Historically, patients in the “vertical” communities each completed custom patient-reported outcome measures tailored to their condition such as the ALS Functional Rating Scale (Revised) [22] in ALS, the MS Rating Scale in MS [23], or a detailed “seizure meter” in epilepsy [20]. Additional condition-specific features included customized visualizations, symptoms, medical history, patient search features, forums, and laboratory tests. Developing and launching these tools was time-consuming, ranging from 3 to 12 months of development for each community. Given the large unmet need (and a waiting list of some 30,000 individuals requesting we build new communities), PatientsLikeMe made major changes to the platform in April 2011, described previously [24,25]

**Figure 1:**
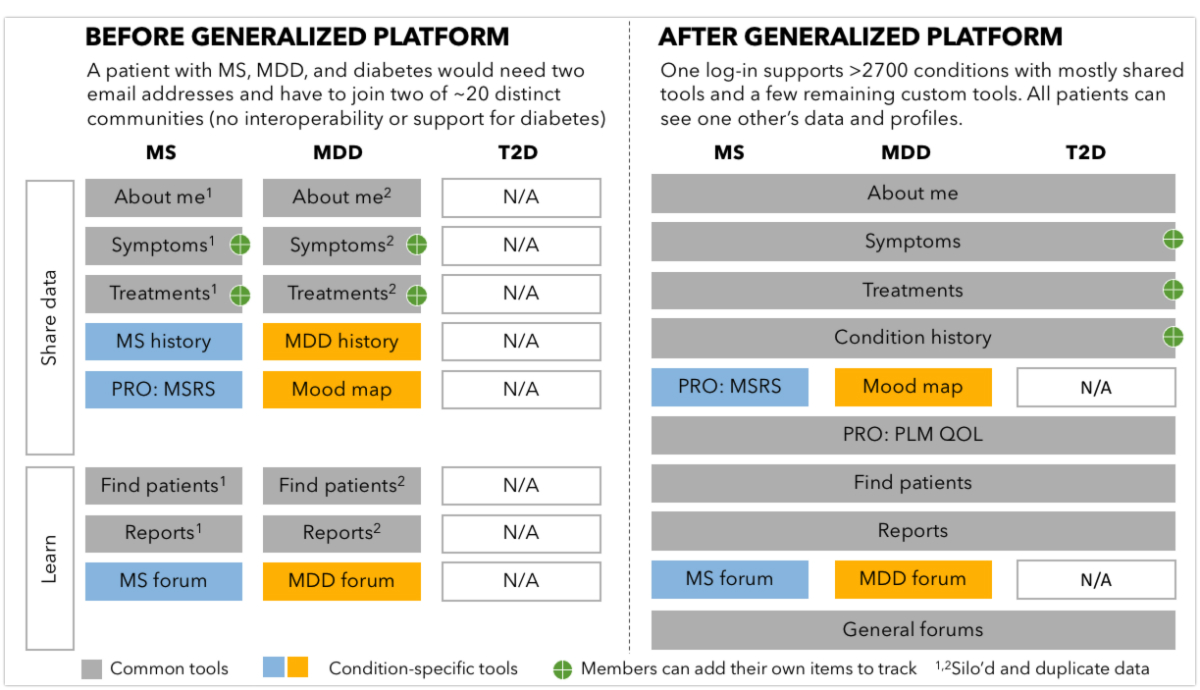
Experience of a hypothetical user before and after platform generalization. Multiple sclerosis (MS) and major depressive disorder (MDD) have community upgrades whereas type 2 diabetes (T2D) does not. MSRS: multiple sclerosis rating scale; N/A: not applicable; PLM QOL: PatientsLikeMe Quality of Life Questionnaire; PRO: patient reported outcome.

To allow any patient to join the site and to track multiple conditions, we developed a more scalable “generalized platform.” This allowed any patient to use a generic quality-of-life outcome measure [26], symptom tracking, treatment tracking, and social networking tools. Members of the generalized platform would not benefit from customized visualizations or a dedicated forum; for instance, and we did not hand-curate condition-specific symptoms or treatments (see Figure 1). However, members could still opt to track their own self-selected symptoms and treatments.

Resulting in part from the generalization of the platform, the site has more than 600,000 registered members across more than 2900 conditions (as of February 2018). However, to date no research has investigated the extent to which members engaging in the general platform experience benefits.

In the years following 2011, a small number of communities benefited from additional “community upgrades” supported by pharmaceutical partnerships. These added site features that would once have only been available to “vertical” communities, such as custom questions, symptoms, laboratory tests, and patient-reported outcomes to communities that would otherwise only have had the generic site functionality. These upgraded communities included psoriasis, idiopathic pulmonary fibrosis, multiple myeloma, and lung cancer.

### Goals of This Study

As a prespecified primary hypothesis, we sought to test whether members of the legacy vertical communities and members with community upgrades would report more benefits than generalized platform members who only had generic tools.

As a secondary prespecified hypothesis, we sought to test whether members who engaged more with the site would experience more benefits [20].

## Methods

### Recruitment

The invitation to participate was fielded to all registered members aged 18 years and older, except those who had opted out of research. Eligible members were invited via email or private message. Users who took no action within 3 days or who started but did not complete the survey were sent a reminder message. Patients were not compensated for participation. Presentation of response rate information including view rate, participation rate, and completion rate, are provided based on Checklist for Reporting Results of Internet E-Surveys (CHERRIES) [27]. This study was reviewed by the New England Institutional Review Board (NEIRB# 16-117) and, as a minimal risk survey, was exempted from further review.

Due to a technical error, some individuals were sent more than one invitation; these were only counted once. If they accidentally completed the survey twice, the latter was excluded. All profile data submissions were optional, so some demographic data are missing.

### Patient-Perceived Benefits of PatientsLikeMe Participation

A custom cross-sectional, retrospective survey was developed to update and build on previous work [19,20]. Level of patient activation was assessed using a validated instrument, the Patient Activation Measure (PAM) [28]. The 13-item validated PAM assesses a consumer’s knowledge, skills, and confidence for self-management. It groups people into one of four levels of activation (1: disengaged and overwhelmed; 2: becoming aware but struggling; 3: taking action; 4: maintaining behaviors and pushing further). Participants were also asked about their recent health utilization (eg, emergency department visits in the past year), general health perception (SF-1), and physician satisfaction. Respondents were asked to estimate how many peers were in their network before and after joining PatientsLikeMe. All survey questions are shown in Multimedia Appendix 1. Demographic data for survey responders and nonresponders were obtained from existing member profile data.

### Statistical Analysis

#### Data Exclusion and Disease Categories

Analysis was limited to patients reporting a physician’s diagnosis of a given condition. Patients were asked to designate a primary condition on their PatientsLikeMe profile; however, they were not required to do so. The median number of comorbidities is reported along with a frequency distribution of those conditions patients considered to be primary. For descriptive and summary purposes, conditions were characterized into therapeutic areas (see Multimedia Appendix 2).

A binary “community customization” variable indicated whether a community had been upgraded with additional functionality and features beyond the general platform experience, regardless of whether it had been a legacy vertical community or benefited from a community upgrade.

#### Reported Benefits in Relation to Site Use

Length of time patients had been registered at the time of survey (“tenure”) was used as a covariate. Three site engagement metrics were generated: (1) number of days spent on the PatientsLikeMe platform (“sessions”), (2) number of days patients conducted social activity on the site (“social”), and (3) whether they had donated any structured data to the platform (“data donation”). Each metric was collapsed into highest and lowest quartiles.

#### Descriptive Statistics

Descriptive statistics were calculated across all survey questions and PAM scores. Summary statistics for continuous variables included n, mean, standard deviation, and range. Median and range were generated for nonnormally distributed variables. Categorical variables were summarized as frequencies and percentages. Patient-perceived benefit questions included a “does not apply” response option because some questions were not relevant to patients (eg, changing treatment if they were not receiving treatment), which were reviewed and removed from percent calculations.

The PAM scores were calculated according to recommended scoring guidelines from the scale developers, including the four levels of activation [25]. The frequency distributions of 15 custom benefit questions were reviewed among the total survey population. A “benefits score” was generated by summing each of the 15 benefits coded as 1 (present) versus 0 (absent).

Chi-square test of independence tested for associations between categorical variables, two-sample *t* tests compared groups on normally distributed continuous variables, with Wilcoxon-Mann-Whitney tests for nonparametric comparisons. One-sample equivalents were performed to compare benefits observed for select conditions to the overall sample. Number of benefits within the overall survey population was stratified based on the presence or absence of community customization, and *P* values adjusted for multiple comparisons using Bonferroni correction.

#### Statistical Models

Separate univariate models assessed the relationship between the dependent variable (number of benefits reported or benefits score) and each independent variable including age, gender, site engagement metrics, patient activation, tenure, and community customization. A review of the sessions and social site metric variables indicated a high correlation and the sessions variable was retained for the final model. All independent variables shown to be significant in the univariate analyses were included in the final multivariable model.

Each model run (univariate and multivariable) was specified as a log-binomial model to estimate the “benefits score” accounting for the independent variables described previously. The response variable “benefits score” was specified as the ratio of events per trials; that is, the number of benefits reported out of the total number of benefits (out of 15). For interpretation purposes, results multiplied by the inverse of this ratio (15) can be provided as the estimate (least square means) of the count of benefits. Relative risks (RRs) were estimated and presented in this model using estimate statements for the appropriate contrasts. An RR greater than 1 is interpreted as a greater chance (“risk”) of an additional benefit compared to the reference category for a given independent variable. The alpha level was set to .05 and all analyses were run in SAS 9.4 by authors ET and CC.

## Results

### Recruitment

**Figure 2:**
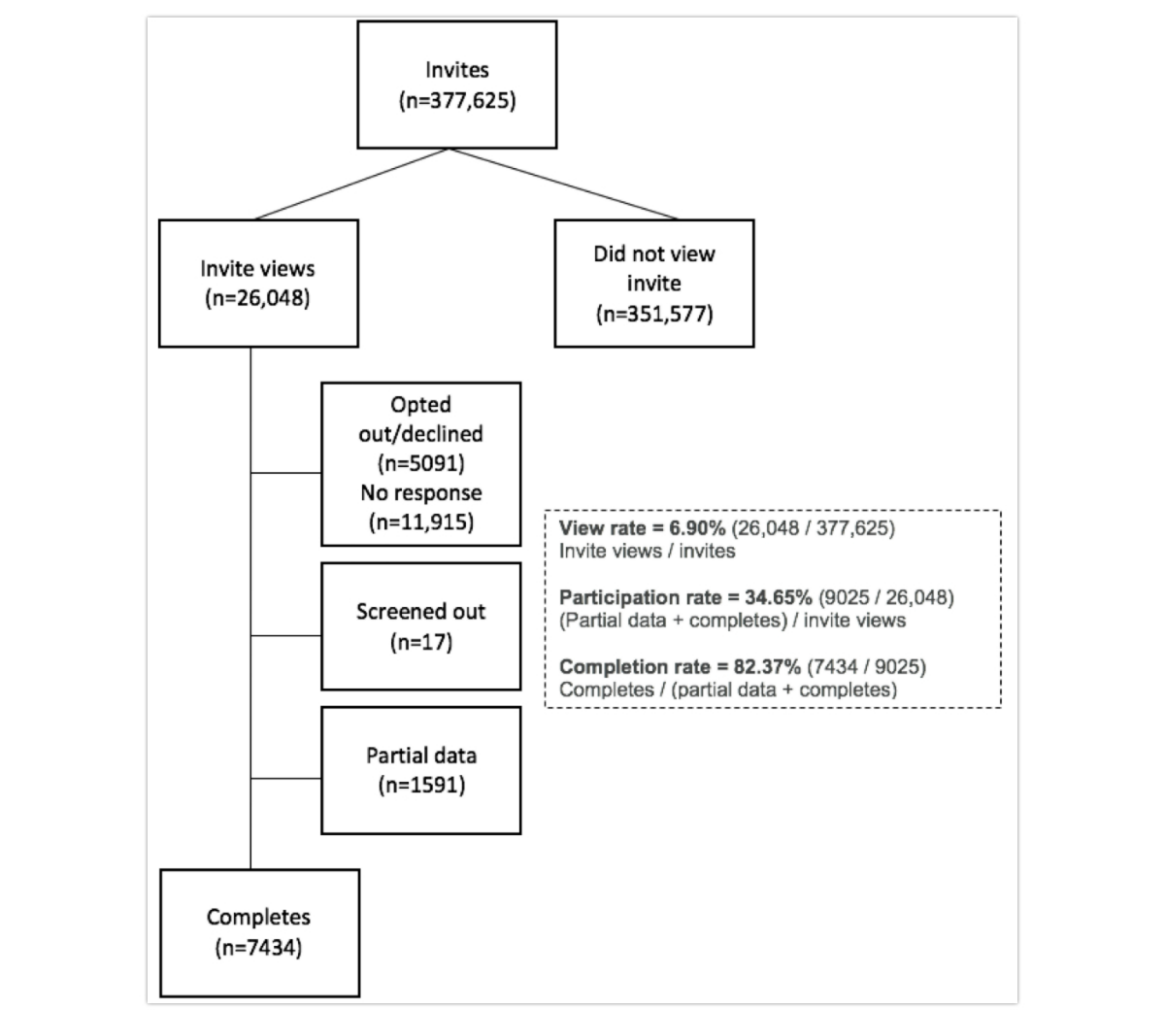
Participant flow.

A total of 377,625 invitations were sent to PatientsLikeMe members between April 11, 2016 and June 20, 2017. By study close, 26,048 of the 377,625 (6.90%) sent an invitation opened the email or private message. Of 26,048 potential respondents who viewed the invitation, 9025 (34.65%) began the survey and 7434 (82.37%) of these completed the survey (Figure 2). Respondents (N=7434) were most frequently white (6106/7052, 86.59%), female (5290/7349, 71.98%), and educated (5062/6026, 84.00% at least some college). Nonresponders were younger, less likely to be white, less educated, and less likely to be on Medicare (see Table 1). Due to a technical error, some individuals were sent more than one invitation; these were only counted once. If they accidentally completed the survey twice, the latter was excluded. All profile data submission was optional, so some demographic data may be missing.

Most patients characterized themselves as being in “fair” or “good” health (5163/7434, 69.45%) and were seeing a specialist (4456/7434, 59.94%; Table 2). Most respondents had at least some level of difficulty with medication adherence (4628/7434, 62.25%). Patient activation was distributed bimodally with the largest percentage at level 3 (2639/7434, 35.50%), and a second peak at level 1 (1858/7434, 24.99%; Table 3).

### Patient-Perceived Benefits of PatientsLikeMe Participation

Most respondents agreed PatientsLikeMe has furthered their understanding of how their condition could affect them (4530/6770, 66.91%) and how to live better with their condition (4247/6750, 62.92%; Table 4). A greater proportion of patients with community customization reported benefits relating to knowledge and understanding of their condition and this was consistently higher than from those in communities without customization (all *P*<.001; Table 4.). Benefits related to positive changes in their condition management and treatment were endorsed at lower rates and showed more variability (Table 4). However, the only benefits for which the enhanced communities did not achieve a significantly greater benefit than the generic communities were better conversations with a health provider, managing symptoms better, stopping a treatment, and changing doctors.

The mean number of benefits reported overall was 6.20 (SD 4.25) out of a possible total of 15 benefits, with a median of 7 (IQR 2-10). A total of 447 primary conditions were represented among survey respondents, with the top 20 conditions shown in Table 5 and across all conditions in Multimedia Appendix 3. The mean count of comorbid conditions in this survey population was 3.65 (SD 4.87) conditions, with a median of 2 (IQR 1-4). Additional analysis of the mean and median benefits for each condition (irrespective of primary condition) is presented in Multimedia Appendix 4 (n=1657 conditions represented in the Impact Survey, with 189 conditions having ≥30 patients reporting).

### Reported Benefits in Relation to Site Use

The mean number of benefits reported was significantly higher among the group with community customization than those without (mean 6.57, SD 4.09 vs mean 5.60, SD 4.43; *t*_4164_=–8.02, *P*<.001) and a similar observation was made considering the median number of benefits (median 7, IQR 3-10 vs median 6, IQR 1-9; Kruskal-Wallis χ^2^_1_=67.3, *P*<.001).

A log-binomial model estimated the number of benefits reported, controlling for community customization, tenure, active sessions, data donation, patient activation, and demographics (Table 6). Results indicated a significant relationship between each independent variable reviewed and the number of benefits reported with the exception of “condition groupings” (Table 6). When all independent predictors were included, the expected number of benefits was 21% higher for members with community customization compared to no community customization (RR=1.21, *P*<.001). The expected number of benefits was 29% higher for patients at the fourth level of activation, compared to the first level (RR=1.29, *P*<.001). This effect was enhanced when limiting the benefits to only those related to knowledge and understanding of one’s disease (42%, RR=1.42), but had less of an effect for those benefits associated with treatment and management of one’s condition (13%, RR=1.13). When a member had a higher level of engagement on the site, the expected number of benefits was 11% higher (RR=1.11, *P*<.001) than for those engaging less. It is important to note that having received benefit from the site may have encouraged respondents to return to the site for more use; however, directionality of engagement could not be directly assessed in this model.

**Table 1:** Demographics of respondents and nonrespondents. IQR: interquartile range.

Characteristic		Responders (n=7434)	Nonresponders (n=370,191)	*P* value^a^
**Age (years), n**		7419	321,886	<.001^b^
	Mean (SD)	54 (12)	48 (14)	
	Median (IQR)	54 (46-62)	48 (38-58)	
**Sex, n (%)**				.61
	Female	5290 (71.16)	232,260 (71.60)	
	Male	2059 (27.70)	92,111 (28.39)	
	Prefer to skip	0	21 (<0.01)	
**Race, n (%)**				<.001
	White	6106 (86.59)	163,857 (80.65)	
	Black	302 (4.28)	9996 (4.92)	
	Mixed race	291 (4.13)	7591 (3.74)	
	Asian	99 (1.40)	8653 (4.26)	
	Native American	90 (1.28)	2175 (1.07)	
	Hawaiian	6 (<0.01)	514 (0.25)	
	Prefer to skip	158 (2.24)	10,397 (5.12)	
**Education, n (%)**				<.001
	High school grad or less	898 (14.90)	18,784 (19.51)	
	Some college	2365 (39.25)	36,172 (37.58)	
	College graduate	1565 (25.97)	22,881 (23.77)	
	Post graduate	1132 (18.79)	15,344 (15.94)	
	Prefer to skip	66 (1.10)	3080 (3.20)	
**Insurance, n (%)**				<.001
	Medicare	1696 (28.03)	15,518 (16.27)	
	Employer	1802 (29.78)	31,994 (33.54)	
	Medicaid	589 (9.73)	8412 (8.82)	
	National	574 (9.49)	10,691 (11.21)	
	Direct	514 (8.49)	8066 (8.46)	
	Veterans Affairs	153 (2.53)	1890 (1.98)	
	Military	115 (1.90)	1729 (1.81)	
	Indian Health Service	2 (<0.01)	108 (0.11)	
	Other	97 (1.60)	1949 (2.04)	
	None	351 (5.80)	8580 (8.99)	
	Prefer to skip	158 (2.61)	6451 (6.76)	

^a^*P* values from *t* test for age and chi-square test for categorical variables.

^b^Satterthwaite method reported due to unequal variances.

**Table 2:** Health utilization and clinical characteristics of survey respondents (N=7434). IQR: interquartile range.

Characteristic		Value
**General health (SF-1), n (%)**		
	Excellent	198 (2.66)
	Very good	968 (13.02)
	Good	2596 (34.92)
	Fair	2567 (34.53)
	Poor	1105 (14.86)
**Physician type, n (%)**		
	A primary care physician	2325 (31.28)
	An internist at a hospital	101 (1.36)
	A specialist in my condition	4456 (59.94)
	I don’t see a physician	278 (3.74)
	I don’t know	183 (2.46)
	Prefer to skip	91 (1.22)
**Treatment satisfaction, n (%)**		
	Extremely dissatisfied	433 (5.82)
	Very dissatisfied	527 (7.09)
	Dissatisfied	1013 (13.63)
	Somewhat satisfied	2326 (31.29)
	Satisfied	1537 (20.68)
	Very satisfied	817 (10.99)
	Extremely satisfied	397 (5.34)
	Does not apply	384 (5.17)
**Health utilization past 12 months, mean (SD), median (IQR)**		
	Emergency department visits	1.26 (3.52), 0 (0-1)
	Overnights in the hospital	2.41 (10.66), 0 (0-1)
	Separate hospital stays	1.26 (6.97), 0 (0-1)
**Adherence (how often do you have difficulty remembering to take your medications?), n (%)**		
	Never/rarely	2614 (35.16)
	Once in a while	2211 (29.74)
	Sometimes	1608 (21.63)
	Usually	514 (6.91)
	All the time	295 (3.97)
	Does not apply	192 (2.58)

**Table 3:** Patient Activation Measure (PAM) distribution among survey respondents (N=7434).^a^

PAM level	Description	n (%)
Level 1	Does not feel in charge of their own health and care. Managing health is overwhelming for them with all of life’s other challenges. Lacks confidence in their ability to manage health. Has few problem-solving skills and poor coping skills. They may not be very aware of own behavior.	1858 (24.99)
Level 2	May lack basic knowledge about their condition, treatment options, and/or self-care. Have little experience or success with behavior change. Look to their doctor to be the one in charge. Low confidence in their ability to manage health.	1449 (19.49)
Level 3	Have the basic facts of their conditions and treatments. Some experience and success in making behavioral changes. Some confidence in handling limited aspects of their health	2639 (35.50)
Level 4	Have made most of the necessary behavior changes, but may have difficulty maintaining behaviors over time or during times of stress	1312 (17.65)

^a^A total of 176 individuals are missing due to selection of the “I prefer to skip” option.

**Table 4:** Perceived benefits among all survey respondents (N=7434).

Benefit		Respondents, n (%)						*P* value^a^
		Total survey respondents (N=7434)^b^		With community customization (n=5344)		Without community customization (n=2045)		
**Knowledge and understanding of condition (Has PatientsLikeMe improved your understanding of...)**													
	How your condition(s) might affect you?	4530 (66.91)	2987 (71.05)		918 (59.11)		<.001	
	What might help you live better with your condition(s)?	4247 (62.92)	2805 (66.77)		852 (54.97)		<.001	
	Treatment side effects?	4182 (60.59)	2747 (64.20)		854 (53.58)		<.001	
	Available treatments?	4143 (60.06)	2765 (64.69)		819 (51.32)		<.001	
	Important factors in making decisions about treatments?	3919 (57.52)	2538 (60.40)		808 (51.04)		<.001	
	What might help you get better?	3339 (50.37)	2159 (52.76)		664 (43.40)		<.001	
	How to deal with other problems in your life (eg, stress, work, money) that may be caused by your condition(s)?	3250 (47.86)	2102 (49.94)		679 (43.30)		<.001	
**Condition treatment and management (As a result of PatientsLikeMe have you...)**												
	Had better conversations with your health care professionals?	3592 (51.62)	2344 (54.61)		762 (47.24)		<.001	
	Managed your symptoms better?	3179 (45.64)	2131 (49.51)		634 (39.28)		<.001	
	Been better at taking your medication?	2251 (33.74)	1437 (34.94)		475 (30.55)		.14	
	Tried a new way to manage side effects?	2089 (29.79)	1374 (32.73)		440 (27.01)		<.001	
	Asked to see a specialist doctor?	1699 (24.79)	1011 (24.06)		411 (25.77)		>.99	
	Started a new treatment?	1046 (14.71)	692 (15.77)		196 (11.90)		.02	
	Stopped a treatment?	993 (13.91)	651 (14.80)		220 (13.27)		.09	
	Changed your doctor?	899 (12.59)	546 (12.38)		220 (13.34)		>.99	

^a^*P* values adjusted using Bonferroni correction.

^b^Percentages calculated out of valid nonmissing data and after removal of “does not apply” responses. A range of approximately 3% to 10% were observed across benefit questions.

**Table 5:** Distribution of conditions most frequently represented in the survey, ordered by condition group then primary condition (N=6264).^a^ IQR: interquartile range.

Patient-reported primary condition	Condition group	n (%)	Mean (SD)	Median (IQR)
Diabetes type 2	Cardiovascular and metabolic diseases	174 (2.78)	6.3 (3.9)	7 (3-9)
Fibromyalgia	Fibromyalgia (other)	668 (10.66)	6.5 (4.0)	7 (3-10)
Crohn’s disease	Inflammation and autoimmunity	49 (0.78)	5.0 (4.2)	5 (1-8)
Rheumatoid arthritis	Inflammation and autoimmunity	128 (2.04)	6.4 (4.1)	7 (3-9)
Systemic lupus erythematosus	Inflammation and autoimmunity	271 (4.33)	6.0 (4.0)	6 (2-9)
Bipolar disorder	Mental and behavioral health	245 (3.91)	6.1 (4.1)	7 (2-10)
Bipolar I disorder	Mental and behavioral health	96 (1.53)	6.9 (4.5)	7 (3-10)
Bipolar II disorder	Mental and behavioral health	130 (2.08)	6.2 (4.0)	7 (3-10)
Complex post-posttraumatic stress disorder	Mental and behavioral health	54 (0.86)	6.5 (4.4)	7 (3-10)
Major depressive disorder	Mental and behavioral health	308 (4.92)	5.7 (3.9)	6 (2-8)
Posttraumatic stress disorder	Mental and behavioral health	232 (3.70)	6.4 (4.1)	7 (3-9)
Traumatic brain injury	Mental and behavioral health	122 (1.95)	6.3 (4.3)	7 (3-10)
Myalgic encephalomyelitis/chronic fatigue syndrome	Myalgic encephalomyelitis/chronic fatigue syndrome (other)	62 (0.99)	5.1 (4.0)	5 (1-8)
Amyotrophic lateral sclerosis	Neurologic	281 (4.49)	5.8 (3.6)	6 (3-9)
Epilepsy	Neurologic	109 (1.74)	7.2 (4.5)	8 (4-11)
Multiple sclerosis	Neurologic	1005 (16.04)	6.5 (3.9)	7 (3-10)
Parkinson’s disease	Neurologic	468 (7.47)	6.2 (4.1)	7 (3-9)
Lung cancer	Oncology	98 (1.56)	5.8 (4.0)	6 (3-9)
Multiple myeloma	Oncology	93 (1.48)	4.9 (3.9)	5 (1-8)
Idiopathic pulmonary fibrosis	Respiratory	150 (2.39)	6.2 (3.8)	6 (3-9)

^a^Primary condition not reported and/or profile data unavailable for n=1170 patients.

**Table 6:** Site use-related predictors of patient-perceived health benefits in the Impact Survey: univariate and multivariate models. Ref: reference group.

Independent variables		Univariate models			Multivariate models				
		Dependent variable: total number of benefits (out of 15)			Dependent variable: total number of benefits (out of 15)			Benefits related to knowledge/understanding of one’s condition (out of 7)	Benefits related to treatment/management of one’s condition (out of 8)
		β	*P*	RR^a^	β	*P*	RR^a^	RR	RR
**Age (years)^b^**									
	18-39	0.12	<.001	1.13	-0.02	0.10	0.98	1.00	0.97
	40-55	0.09	<.001	1.10	-0.14	<.001	1.12	0.91	0.85
	≥55	0	—	Ref	0	—	Ref	Ref	Ref
**Gender**									
	Male	0	—	Ref	0	—	Ref	Ref	Ref
	Female	0.07	<.001	1.08	0.07	<.001	1.07	1.04	1.16
Tenure at baseline^c^		0.02	<.001	1.02	0.02	<.001	1.02	1.02	1.02
**Community customization**									
	None	0	—	Ref	0	—	Ref	Ref	Ref
	Customized	0.16	<.001	1.17	0.19	<.001	1.21	1.24	1.13
**Patient activation**									
	Level 1	0	—	Ref	0	—	Ref	Ref	Ref
	Level 2	0.15	<.001	1.16	0.14	<.001	1.15	1.21	1.06
	Level 3	0.21	<.001	1.24	0.21	<.001	1.23	1.32	1.12
	Level 4	0.25	<.001	1.29	0.25	<.001	1.29	1.42	1.13
**Sessions**									
	Low engagement	0	—	Ref	0	—	Ref	Ref	Ref
	Mod-high engagement	0.13	<.001	1.14	0.10	<.001	1.11	1.05	1.19
**Condition grouping**									
	Cardiovascular and metabolic diseases	0.04	.10	1.04	0.18	<.001	1.19	1.22	1.07
	Infectious disease	–0.09	.08	0.91	–0.09	.08	0.91	0.87	0.98
	Inflammation and autoimmunity	–0.04	.03	0.96	–0.01	.52	0.99	1.03	0.89
	Mental and behavioral health	0.07	<.001	1.07	0.05	.002	1.05	1.03	1.05
	Neurologic	0.07	<.001	1.07	0.01	.58	1.01	1.08	0.89
	Oncology	–0.19	<.001	0.82	–0.07	.006	0.93	1.03	0.74
	Respiratory	0.05	.07	1.05	0.10	.002	1.10	1.15	1.03
	Other	0	—	Ref	0	—	Ref	Ref	Ref

^a^Relative risk (RR) greater than 1 represents a greater chance (“risk”) of an additional benefit compared to the reference category.

^b^Representation of the survey population in the category 18-24 years was quite low (2%) and thus was combined with the category 25-39 years to create the 18-39 years category. As seen in Table 1, the mean age in the population was 54.

^c^Tenure at baseline variable converted to years (from days) for interpretation purposes.

## Discussion

### Principal Results

This study confirmed that members joining PatientsLikeMe in the “generalized platform” do perceive a variety of benefits from their participation on the site. The majority of members learned more about how their condition might affect them, what might help them live better with their condition, how to manage treatment side effects, to be more aware of existing treatments, and had an improved understanding of what might help them get better. Although the average member perceived around six of a possible 15 benefits from using the site, this increased to seven benefits in conditions that had some degree of community customization, confirming our primary hypothesis. Within members of a “customized” condition, benefits were higher for the most engaged members who logged in, socialized, or entered data the most frequently, partially confirming our secondary hypothesis (the effect was absent for those without customization).

It is worth considering whether the extra design, research, testing, and coding that goes into customizing a community is worth the increase of a single reported benefit. Of the 2700 or so communities represented on the platform, the vast majority have not received site customization and yet perceive a similar number of benefits. This may reflect some “floor effect” where the basic functionality of the site, which permits any member to record their health-related quality of life, connect with others, and optionally track their own self-selected symptoms and treatments, is already doing a reasonable job at fulfilling a patient’s needs. One advantage of additional community customization has been the addition of outcome measures of clinical relevance, such as oxygen use in idiopathic pulmonary fibrosis or body surface area coverage in psoriasis. Future developments on the site aim to make it much simpler (and therefore more scalable) to permit some degree of community customization through simple changes made on an “admin dashboard” rather than requiring new code. We also plan to develop and psychometrically validate a modular patient-reported outcome system that can then be validated clinically against gold standard measures in various conditions.

### Limitations

There are several limitations of this study that are important to consider. Although putting the response rates of surveys in online communities into perspective is not straightforward [27], the responses provided here represent just 2% of the whole community. However, that denominator includes patients who registered a decade ago, some of whom may be deceased, too disabled to participate, or who may even have recovered to some extent from their condition. The finding that the most active ~1% of online community membership is responsible for much of its positive impact has been observed in other online health communities [15].

Given the cross-sectional design, directionality of benefits and site use characteristics could not be discerned. Although the extent of customization for communities varies, this level of granularity was not reviewed in favor of a simpler indicator. The benefits reported were self-reported with no independent validation and no control group. Furthermore, due to the potential selection bias, social desirability bias, and given that the data represent a convenience sample, the results are not generalizable to the overall population, particularly given the skew toward a more educated, female population. Another limitation is the complexity of real-world data as it relates to comorbid conditions. To minimize complexity, condition-specific data was limited to those patients who had reported a primary condition and indicated a diagnosis of this condition on the site. However, not all patients report a primary condition, this designation may change over time, and the restriction to a primary condition does not account for the reality of complex, comorbid conditions.

Some comparisons presented in the study were not all specified a priori and are presented as exploratory results. Finally, although receiving more benefits from having participated on the site is preferable to fewer benefits, it is also possible that having received even one benefit, such as having better conversations with your health care provider or starting a new treatment, is meaningful for a patient. No weight was given to one benefit over another.

It may be interesting in future studies to evaluate combinations of benefits or to explore latent class characterizations of the latent categories or typologies of benefits.

### Comparison With Prior Work

Overall, these findings align with earlier studies reported in the literature [19,20], although due to questions being asked differently it is not possible to compare benefits like-for-like. Strengths of this study include the relatively large sample size and the diversity of conditions represented, from mood disorders to infections to neurological conditions to oncology. Current literature shows that patients using other online communities benefit too, but studies have generally been limited to emotional benefits in a few communities for specific conditions or a study of one specific disease state, and often as part of an interventional education program rather than peer-to-peer communication.

A study of Reddit forums for depression found that engagement was linked to more positive emotional states and an increased use of positive words [29]. A study in the United States found that women with breast cancer who participated in a 12-week Internet-based social group reported reduced depression, stress, and cancer-related trauma [30]. Additionally, a study in Japan also focused on breast cancer patients found that online support groups provided benefits to participants, but patients who posted in the forums felt they received more benefits than the “lurkers” did [31]. This study uniquely adds to the literature in that PatientsLikeMe has been open to all patients since 2011. The main benefits reported reflect the intent of the website, which is to help patients find other patients like them, track their own personal health data over time to discover insights about their health, and to share their experiences to benefit others.

Benefits reported at lower rates, such as managing treatment side effects, asking to see a specialist doctor, or changing doctor, do not have any specific tools or functionality assigned to them on the site. Such changes in health behavior may arise from serendipitous social interactions with other members in community spaces, increased awareness of treatment options and coping methods identified on treatment reports, or increases in patient activation as members become more aware of their bodies and health state.

Findings also support PatientsLikeMe’s business model. Although imperfect, other business models such as advertising or asking members to pay a subscription fee have their own challenges. Relying entirely on grant income presents a challenge to stability and continuity for an enterprise that has been operating for over a decade. The number of benefits reported by patients identifying with conditions for which there was community customization and patient-centric research was 18% higher than for patients reporting conditions without this additional functionality and opportunity. From 2011 to the present, the majority of investment in site customization and patient-centric research has come from PatientsLikeMe partnerships with pharmaceutical companies such as AstraZeneca [9] and research grants from funders such as the Robert Wood Johnson Foundation. This study therefore demonstrates that commercial partnerships may have a positive impact on members who might not otherwise have found the site, while simultaneously enabling partners to learn about patient priorities for new therapies and implement patient-centric programs such as improving their clinical trials [32].

It is also important to note that although community customization was one factor associated with benefits, patient factors were important too, such as engagement and activation. Previous research on patient activation has indicated that 41% of US adults are characterized as being in the highest level of activation (level 4), with approximately 7% at level 1 and 15% at level 2 [33]. Lower activation has been found in those with low income, less education, Medicaid enrollees, and people with poor self-reported health [33]. Patient respondents in this survey were less activated than the general population, perhaps reflecting the higher burden they face as a result of their chronic conditions, or identifying the reason they might have sought the assistance of an online community in the first place.

### Future Research

To further elucidate who benefits most from participation in the site, a study is currently being conducted to evaluate benefits over time (6 weeks) with a pre-post longitudinal study design to explore patterns in patient-perceived benefits as well as changes in patient activation overall and by conditions.

### Conclusion

Online health communities offer an opportunity for patients to connect and share information that provides benefits in their health management. Generalized health communities offer benefits to users and partnering with stakeholders in patient health to enhance and customize these communities is one way to offer greater benefits to patients.

## Appendix

Multimedia Appendix 1 Impact survey questionnaire.

Multimedia Appendix 2 List of "other" conditions.

Multimedia Appendix 3 Benefits for all primary conditions.

Multimedia Appendix 4 Benefits for all primary and secondary conditions.

## Abbreviations

ALS: amyotrophic lateral sclerosis
CHERRIES: Checklist for Reporting Results of Internet E-Surveys
IQR: interquartile range
MS: multiple sclerosis
MDD: major depressive disorder
MSRS: multiple sclerosis rating scale
PAM: Patient Activation Measure
PLM QOL: PatientsLikeMe Quality of Life Questionnaire
PRO: patient reported outcome
RR: relative risk
T2D: type 2 diabetes
