# Supplementary material for: Scaling PatientsLikeMe via a “Generalized Platform” for Members with Chronic Illness: Web-Based Survey Study of Benefits Arising
Source: J Med Internet Res. 2018 May 7;20(5):e175. doi: 10.2196/jmir.9909 (PMC5962830; doi:10.2196/jmir.9909)
Supplement: Multimedia Appendix 1 [file jmir_v20i5e175_app1.pdf]

Multimedia Appendix A – Impact Survey Questionnaire.

Impact Survey Questionnaire

Screener

Are you age 18 or over?

- Yes
- No [screen out]

Health, Utilization, and Behaviors (5Q's)

1. In general, would you say your health is: (Excellent, very good, good, fair, poor)
2. During the past 12 months, how many times did you go to the emergency room (ER) of a hospital for examination or treatment related to your condition(s)? (Number)
3. During the past 12 months, how many nights did you stay overnight in a hospital for examination or treatment related to your condition(s)? (Number)
4. During the past 12 months, how many separate overnight stays did you have in a hospital for examination or treatment related to your condition(s)? Please count each stay separately whether you stayed one night or more. (Number)
5. How often do you have difficulty remembering to take all your medications? (Never/rarely, once in a while, sometimes, usually, all the time, does not apply)
6. In general, how satisfied or dissatisfied are you with the ability of the medication you take to prevent or treat your condition(s)? (Extremely dissatisfied, very dissatisfied, dissatisfied, somewhat satisfied, satisfied, very satisfied, extremely satisfied, does not apply)

Provider Information (3Q's – skip logic: only show this section to patients in the US and insurance)

7. Do you have a health plan with a high deductible? (i.e. more than $1,300 for an individual or $2,600 for a family) (Yes, no, I don't know, does not apply to me, I prefer not to say)
8. Did you reach the limit of your health plan deductible last plan year? (Yes, no, I don't know, does not apply to me, I prefer not to say)
9. What kind of physician do you normally see to manage your condition? (A primary care physician (PCP), an internist at a hospital, a specialist in my condition, I don't see a physician, I don't know, I prefer not to say)

Patient Activation Measure (PAM-13).

Below are some statements that people make when talking about their health. Please indicate how much you agree or disagree with each statement as it applies to you personally.

10. When all is said and done, I am the person who is responsible for managing my health condition(s) (Strongly disagree, disagree, agree, strongly agree)
11. Taking an active role in my own health is the most important factor in determining my health and ability to function (Strongly disagree, disagree, agree, strongly agree)
12. I am confident that I can take actions that will help prevent or minimize some symptoms or problems associated with my health condition(s) (Strongly disagree, disagree, agree, strongly agree)
13. I know what each of my prescribed medications do (Strongly disagree, disagree, agree, strongly agree)
14. I am confident that I can tell when I need to go get medical care and when I can handle a health problem on my own (Strongly disagree, disagree, agree, strongly agree)
15. I am confident that I can tell a doctor concerns I have even when he or she does not ask (Strongly disagree, disagree, agree, strongly agree)
16. I am confident that I can follow through on medical treatments that I need to do at home (Strongly disagree, disagree, agree, strongly agree)
17. I understand the nature and causes of my health condition(s) (Strongly disagree, disagree, agree, strongly agree)
18. I know the different medical treatment options available for my health condition(s) (Strongly disagree, disagree, agree, strongly agree)
19. I have been able to maintain the lifestyle changes for my health condition(s) that I have made (Strongly disagree, disagree, agree, strongly agree)
20. I know how to prevent further problems with my health condition(s) (Strongly disagree, disagree, agree, strongly agree)
21. I am confident that I can figure out solutions when new situations or problems arise with my health condition(s) (Strongly disagree, disagree, agree, strongly agree)
22. I am confident that I can maintain lifestyle changes, like diet and exercise, even during times of stress (Strongly disagree, disagree, agree, strongly agree)

Insignia Health. “Patient Activation Measure; Copyright © 2003-2010, University of Oregon. All Rights reserved.” Contact Insignia Health at www.insigniahealth.com

Improved knowledge/understanding (8Q’s)

PatientsLikeMe aims to help you better understand and manage your health as well as get support from others like you. Help us understand how we're doing. Has PatientsLikeMe improved your understanding:

23. of how your condition(s) may affect you? (Yes/No/Does not apply)
24. of what might help you live better with your condition(s)? (Yes/No/Does not apply)
25. of what might help you get better? (Yes/No/Does not apply)
26. of available treatments? (Yes/No/Does not apply)
27. of treatment side effects? (Yes/No/Does not apply)
28. of the important factors in making decisions about treatments? (Yes/No/Does not apply)
29. of how to deal with other problems in your life (e.g. life, work, money) that may be caused by your condition(s)? (Yes/No/Does not apply)

Improved Condition Management and Treatment (8Q’s)

PatientsLikeMe aims to help you better understand and manage your health as well as get support from others like you. Help us understand how we're doing. As a result of using PatientsLikeMe have you:

30. Started a new treatment? (Yes/No/Does not apply)
31. Stopped a treatment? (Yes/No/Does not apply)
32. Tried a new way to manage reduce side effects? (Yes/No/Does not apply)
33. Been better at taking your medication? (Yes/No/Does not apply)
34. Managed your symptoms better? (Yes/No/Does not apply)
35. Had better conversations with your healthcare professionals? (Yes/No/Does not apply)
36. Changed your doctor? (Yes/No/Does not apply)
37. Asked to see a specialist doctor? (Yes/No/Does not apply)

Improved network (5Q's)

38. Before joining PatientsLikeMe, how many other patients with the same condition(s) as you had you communicated with? (number)

39. Since joining PatientsLikeMe, how many other patients with the same condition(s) as you have you communicated with? (number)

NPS for PLM

40. How likely is that you would recommend PatientsLikeMe to a friend or family member?
○ 0-10 Horizontal radio buttons (0- Not at all likely, 10 - Extremely likely, I prefer to skip)

Open response

41. Is there anything else you would like to tell us about how PLM has had an impact on you? [Open text]

Standard Demographics

42. When were you born?
- Drop down menus; year (required), month (optional), day (optional)

43. What is your sex
- male
- Female
- I prefer to skip

44. Which of the following types of places best describes where you live?
- Urban area
- Suburban area

- Rural area
- Other

45. What race do you consider yourself to be?
- White
- Black or African American
- Asian
- Native Hawaiian or other Pacific Islander
- American Indian or Alaskan Native
- Mixed Race

46. What ethnicity do you consider yourself to be?
- Hispanic or Latino
- Not Hispanic or Latino
- I prefer to skip

47. What type of health insurance do you have?
- Private (through employer or union)
- Private (individual plan)
- Medicare
- Medicaid (or other low-income government plan)
- Veteran's Administration
- TRICARE (or other military health insurance)
- Indian Health Service
- National health service
- Other type of insurance
- No Insurance

48. What is your highest level of education?
- 8th grade or less (left school around 14)
- Some high school, but did not graduate (left school around 16)
- High school graduate or GED (left school around 18)
- Some college but less than a bachelor's / undergraduate degree
- College bachelor's / undergraduate degree
- Postgraduate degree (Master's, doctorate, etc.)

49. What Country do you live in?
    - Drop down list of countries

50. STATE: What US State do you live in? If you do not live in the US, please select "does not apply"
- Drop-down menu with state list
- [Skip to last question if no state selected]

51. ZIP: What is your zip code? [only if a state is selected]

- Open numeric
