# Supplementary material for: Scaling PatientsLikeMe via a “Generalized Platform” for Members with Chronic Illness: Web-Based Survey Study of Benefits Arising
Source: J Med Internet Res. 2018 May 7;20(5):e175. doi: 10.2196/jmir.9909 (PMC5962830; doi:10.2196/jmir.9909)
Supplement: Multimedia Appendix 2 [file jmir_v20i5e175_app2.pdf]

Multimedia Appendix 2: Condition Categorizations

“Other” Conditions
Fibromyalgia
Myalgic Encephalomyelitis/Chronic Fatigue Syndrome
Chronic Pain Syndrome
Hypothyroidism
Ehlers-Danlos Syndrome
Pompe Disease
Hypogammaglobulinemia
Common Variable Immunodeficiency Disorder
Alkaptonuria
Irritable Bowel Syndrome
Polycythemia Vera
Charcot-Marie-Tooth Disease
Chronic Kidney Disease
Gastroparesis
Herniated Disc
Interstitial Cystitis
Polymyalgia Rheumatica
Spinal Stenosis
Myofascial Pain Syndrome
Polycystic Ovary Syndrome
Addison's Disease
Amlyoidosis
Dermatomyositis
Hypermobility Syndrome
Adrenal Insufficiency
Chiari Malformation
Endometriosis
Failed Back Syndrome
Hereditary Spastic Paraplegia
Meniere's Disease
Osteoporosis
Post-Concussion Syndrome
Primary Biliary Cholangitis
Sarcoidosis
Scoliosis
Spondylolisthesis
Acromegaly Syndrome
Acute Renal Failure
Adrenal Fatigue
Alpha 1 Antitrypsin Deficiency

Antiphospholipid Antibody Syndrome
Atopic Dermatitis
Benign Prostatic Hyperplasia
Cerebral Palsy
Chronic Idiopathic Back Pain
Concussion
Crest Syndrome
Dercum's Disease
Factor V Leiden
Familial Mediterranean Fever
Gastroesophageal Reflux Disease
Hemophilia A
Hiatal Hernia
Igg Deficiency
Inclusion Body Myositis
Irritable Bladder Syndrome
Liver Cirrhosis
Mast Cell Activation Syndrome
Microscopic Colitis
Mitochondrial Myopathy
Non Obstructive Hypertrophic Cardiomyopathy
Nonalcoholic Steatohepatitis
Osteopenia
Pernicious Anemia
Polycystic Kidney Disease
Spinal Cord Injury
Spinocerebellar Ataxia
Syringomyelia
Temporomandibular Joint Syndrome
Alexander Disease
Antisynthetase Syndrome
Aspartylglucosaminuria
Atypical Facial Pain
Bilateral Vestibualar Hypofunction
Brachial Plexus Injury
Broken Femur (Left)
Bullous Pemphigoid
Burning Mouth Syndrome
Car Accident
Carcinoid Syndrome
Chemical/Venom Allergy
Chronic Idiopathic Hives
Congenital Rubella
Congenital Spinal Stenosis

Costochondritis
Cushing's Syndrome
Cystitis Cystica
Diverticulosis
Dry Eye Syndrome
Eosinophilic Esophagitis
Episodic Ataxia
Erectile Dysfunction
Erosive Osteoarthritis
Facioscapulohumeral Muscular Dystrophy
Female Infertility
Foot Stress Fracture
Gastrointestinal Polyps
Glaucoma
Glomerulonephritis
Growth Hormone Deficiency
Hearing Loss
Hereditary Hemorrhagic Telangiectasia
Hereditary Palmoplantar Keratoderma
Hyper Ige Syndrome
Hyperparathyroidism
Hyperthyroidism
Hypertrophic Obstructive Cardiomyopathy
Hypocomplementemic Urticarial Vasculitis Syndrome
Indolent Systemic Mastocytosis
Kartagener Syndrome
Kidney Cyst
Knee Injury
Kyphoscoliosis
Liddle's Syndrome
Limb Girdle Muscular Dystrophy Type 2i
Lumbar Disc Annular Tear
Lumbar Spondylosis
Madelung Deformity
Mass Phenotype
Mastocytic Colitis
Menorrhagia
Methylenetetrahydrofolate Reductase Deficiency
Narcotic Bowel Syndrome
Neurofibromatosis
Neurogenic Bladder
Non-Alcoholic Fatty Liver Disease
Oculomotor Apraxia
Ovarian Cyst

Pancreas Divisum
Panhypopituitarism
Patent Foramen Ovale
Pelvic Floor Dyssynergia
Perennial Allergy
Plantar Fasciitis
Polycystic Liver Disease
Portal Vein Thrombosis
Primary Sclerosing Cholangitis
Retinitis Pigmentosa
Retroperitoneal Fibrosis
Russell-Silver Syndrome
Sexual Abuse
Specific Antibody Deficiency
Spondylosis
Statin-Induced Myopathy
Subacute Thyroiditis
Surgical Menopause
Temporomandibular Joint Disc Dislocation
Tennis Elbow (Lateral Epicondylitis)
Thalassemia Minor
Tinnitus
Torn Meniscus Of Knee
Von Willebrand Disease
